# Scaling-Up of Dental Pulp Stem Cells Isolated from Multiple Niches

**DOI:** 10.1371/journal.pone.0039885

**Published:** 2012-06-29

**Authors:** Nelson F. Lizier, Alexandre Kerkis, Cícera M. Gomes, Josimeri Hebling, Camila F. Oliveira, Arnold I. Caplan, Irina Kerkis

**Affiliations:** 1 Laboratory of Genetics, Butantan Institute, Sao Paulo, SP, Brazil; 2 Departament of Morphology of Federal University of Sao Paulo (UNIFESP), Sao Paulo, SP, Brazil; 3 Department of Orthodontics and Pediatric Dentistry of State University of Sao Paulo (UNESP), Araraquara, SP, Brazil; 4 Skeletal Research Center, Department of Biology of Case Western Reserve University, Cleveland, Ohio, United States of America; University of Freiburg, Germany

## Abstract

Dental pulp (DP) can be extracted from child’s primary teeth (deciduous), whose loss occurs spontaneously by about 5 to 12 years. Thus, DP presents an easy accessible source of stem cells without ethical concerns. Substantial quantities of stem cells of an excellent quality and at early (2–5) passages are necessary for clinical use, which currently is a problem for use of adult stem cells. Herein, DPs were cultured generating stem cells at least during six months through multiple mechanical transfers into a new culture dish every 3–4 days. We compared stem cells isolated from the same DP before (early population, EP) and six months after several mechanical transfers (late population, LP). No changes, in both EP and LP, were observed in morphology, expression of stem cells markers (nestin, vimentin, fibronectin, SH2, SH3 and Oct3/4), chondrogenic and myogenic differentiation potential, even after cryopreservation. Six hours after DP extraction and *in vitro* plating, rare 5-bromo-2′-deoxyuridine (BrdU) positive cells were observed in pulp central part. After 72 hours, BrdU positive cells increased in number and were found in DP periphery, thus originating a multicellular population of stem cells of high purity. Multiple stem cell niches were identified in different zones of DP, because abundant expression of nestin, vimentin and Oct3/4 proteins was observed, while STRO-1 protein localization was restricted to perivascular niche. Our finding is of importance for the future of stem cell therapies, providing scaling-up of stem cells at early passages with minimum risk of losing their “stemness”.

## Introduction

Isolation of stem cells (SC) from human adult and deciduous teeth has been reported in the last decade [1], [2]. In this short period of time, considerable progress has been achieved, in particular, with deciduous teeth stem cells (DTSC) [3]. It has been demonstrated that the use of different handling methods of dental pulp (DP) can lead to the isolation of SC populations with distinct properties. These DTSC populations are similar to mesenchymal stem cells (MSCs) or epithelial SCs or they are composed by a mixed population of both cell types [3]. We previously isolated a population of multipotent DTSCs, which were referred to as “immature” (Immature Dental Pulp Stem Cells, IDPSCs). Along with MSC markers, IDPSCs express embryonic stem (ES) cells markers (Oct3/4, Nanog and Sox2) and undergo spontaneous differentiation into a wide range of cell types *in vitro* [4]. These cells showed expressive capacity to contribute into multiple tissues in response to the cellular milieu during human/mouse pre-termed chimeras development [5]. After transplantation of IDPSCs into different adult animals, including mouse, rabbit and dog, neither immune rejection nor teratoma formation was observed [6], [7], [8]. IDPSCs and other dental stem/progenitor cells were recently used to obtain induced pluripotent SCs [9], [10]. These cells demonstrated higher efficiency of reprogramming than fibroblasts, providing a model for the study of pediatric diseases and disorders. Taken together, these data strongly suggest that IDPSCs are a hopeful source for the future of SC therapies [11].

Recent SC research studies revealed a promising potential of MSCs to treat at least ten human diseases: heart disease, diabetes, Crohn’s disease, deafness, autoimmune disorders, leukemia, cancers, sickle cell disease, amyotrophic lateral sclerosis and metabolic disorders. Since MSCs are present at low relative amounts in bone marrow and other adult tissues, significant *in vitro* expansion is necessary in order to generate sufficient quantities of these cells to treat human disease [12], [13]. The expansion process itself induces senescence of MSCs and loss of their stemness as shown by a decline in proliferative and differentiation capacity [14], [15]. In addition, prolonged culturing of MSCs increases the probability of genetic changes, which could affect their safe use in clinical trials and future therapies [16], [17]. Therefore, studies which provide adequate production of SC of excellent quality at early passages derived from the same donor are of importance.

Our group was the first to use explant culture of DP to obtain DTSCs, which in combination with appropriate cell culture conditions, provides isolation of a relatively pure (not homogeneous) population of IDPSC [4]. Subsequent study demonstrated the advantages of DP explant culture for the differentiation and proliferation potentials of SC [18]. Herein, we aimed to establish a new method based on tissue explant culture and mechanical (non-enzymatic) transfer in order to obtain a long-term culture of DP providing substantial quantities of DTSCs without aberrant genetic and biologic changes. DP was maintained in culture following mechanical transfer during several months. We evaluated such characteristics as: morphology, expression of specific MSC-phenotypes and ES cell proteins and genes, karyotype, growth rate and differentiation ability of IDPSCs just after DP extraction (early population, EP) and after multiple DP transfer (late population, LP). Some of these parameters were evaluated after cryopreservation and with culturing IDPSCs in three distinct culture media. The used of antibody against BrdU incorporated in DP just after plating and three days after DP cultivation gave insight into the mechanism of IDPSCs generation by explant culture. Additionally, to distinguish SCs in DP, immunohistochemical staining against nestin, vimentin, Oct3/4 and STRO-1 has been performed.

## Results

### Long-term Culture of DP

**Figure 1:**
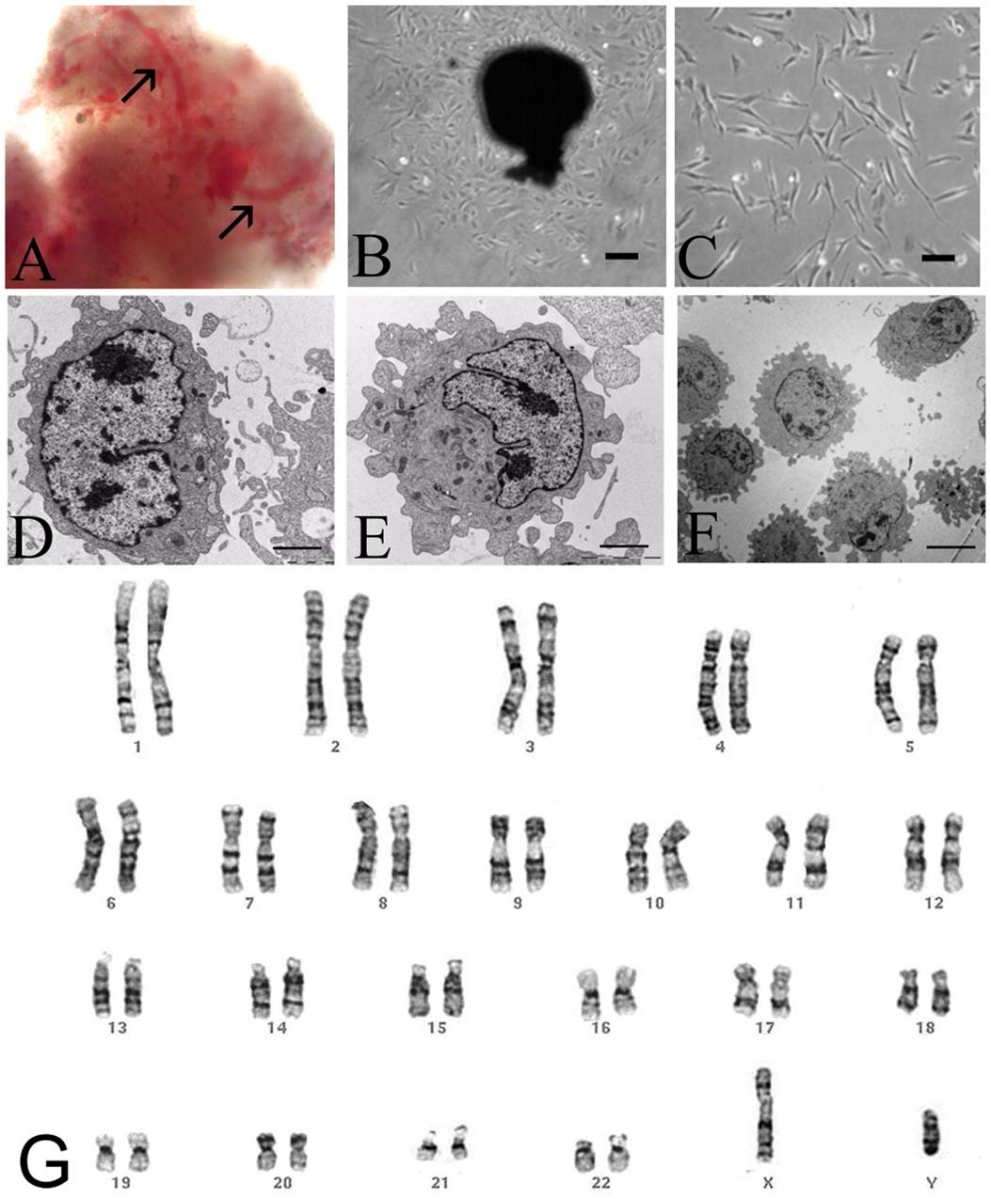
Dental pulp and IDPSCs. **A**) Highly vascularized (black arrows) DP just after extraction. **B**) Explant culture of DP with outgrowing IDPSCs. **C**) Culture of IDPSCs at 1^st^ passage. **D**) IDPSCs showing ES-like cells morphology with a large nucleus. **E**) IDPSCs showing MSC-like morphology with several pseudopodes. **F**) IDPSCs showing uniform morphology resembling ES cells and MSCs. **G**) Karyotype of IDPSCs (LP): chromosomes in pairs, ordered by size and position did not reveal any numerical changes in chromosome number; G-banding analysis. A–C, G) Light Microscopy; D–F) Transmission Electron Microscopy; A = 20X, G = 63X; Scale bars: B = 20 µm; C, F = 10 µm; D, E = 3 µm.

**Figure 2:**
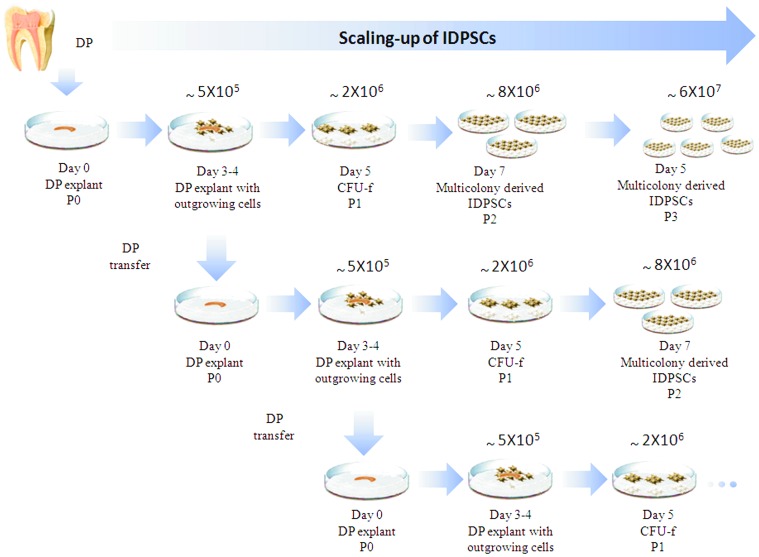
Scaling-up of IDPSCs. Horizontally, the process of DP *in vitro* plating (Day 0, P0) followed by DP adherence and cells outgrowth (Day 3–4). This process is followed by enzymatic treatment (P1) of the cells and formation of multiple colonies (CFU-f - Colony Forming Units-fibroblast). After 5 days, enzymatic treatment was performed to harvest multicolony-derived IDPSCs (P2) population. Next, *in vitro* expansion of IDPSCs (P3) has been performed. Upper numbers represent approximate quantity of harvested IDPSCs in each passage. Vertically, the same process is shown, albeit after multiple DP mechanical transfer.

Freshly extracted DP is a tissue, which contains large nerve trunks and blood vessels in the central region of the coronal and radicular pulp (Figure 1A). First outgrowing fibroblast-like cells appeared between three to four days after DP plating (Figure 1B). Long-term culture was performed by mechanical transfer of DP into new culture dish without using enzymatic treatment. After each transfer, DP produces large numbers of outgrowing cells approximately every three or four days, thus allowing constant production of SCs at passage zero (P0) (Figure 2). We obtained successful isolations with all samples (n = 10) of deciduous teeth. We performed multiple DP transfers during, at least, six months (LP of IDPSCs). Both, EP and LP of IDPSCs maintained their morphology (Figure 1C). Transmission electron microscopy revealed two types of IDPSCs morphology: ES-like cells with low cytoplasm-to-nucleus ratio, low cytoplasm density, which are poor of organelles (Figure 1D). IDPSCs of MSC-like cells have a high number of stretched out pseudopodes, which serve to explore substrate and more cytoplasm and organelles when compared with IDPSCs of ES-like cells (Figure 1D, E). Figure 1F documents that IDPSCs showed a relatively uniform population in respect of these two cell types. IDPSCs karyotype was confirmed here to be unchanged, suggesting that during culture, numerical and gross structural chromosomal abnormalities did not occur as shown by routine G-banding technique (Figure 1G). Further, *in vitro* expansion of IDPSCs was performed using enzymatic treatment and the passages was called P1 (Figure 2).

### Immunophenotyping of EP and LP of IDPSCs

**Figure 3:**
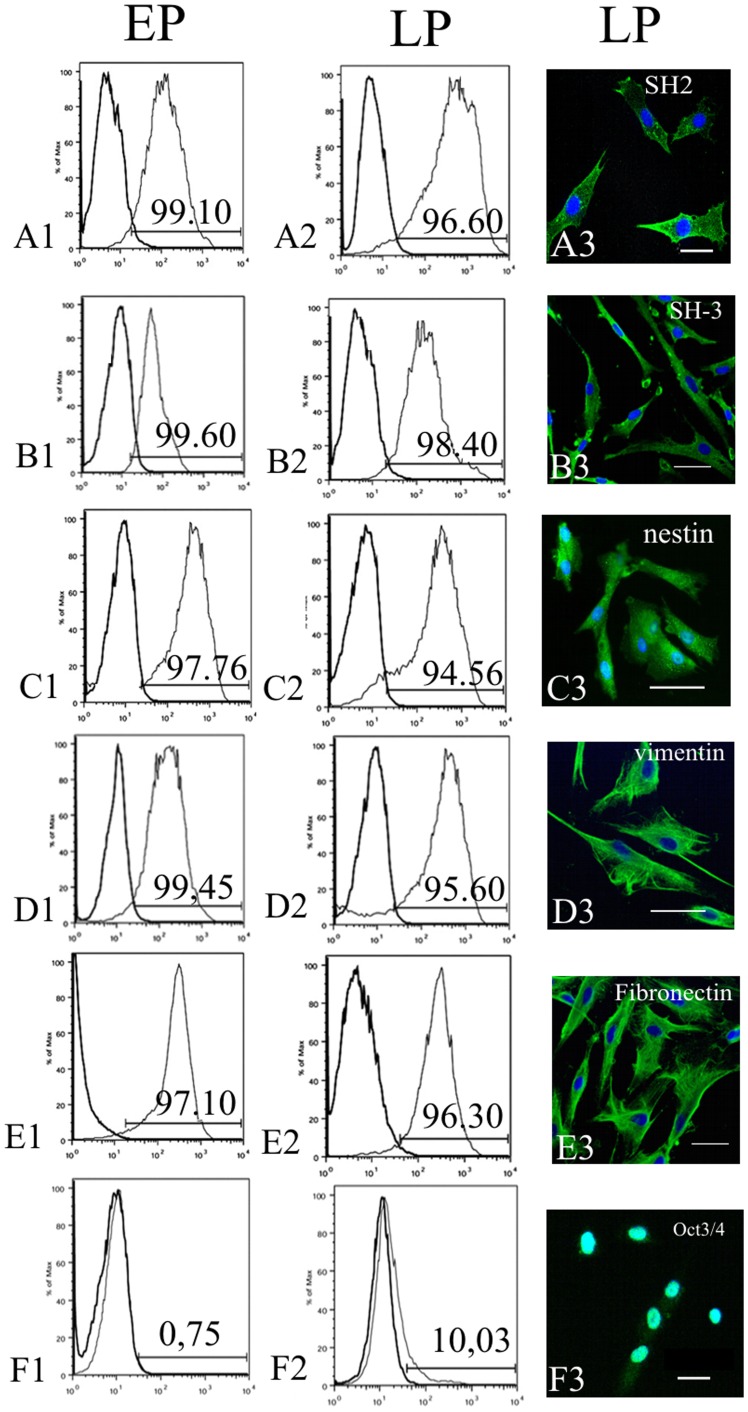
Characterization of EP and LP of IDPSCs. **A1–F1)** Flow cytometry showing EP of IDPSCs, which highly expressed such markers as SH2/CD105 (A1); SH3/CD73 (B1); nestin (C1); vimentin (D1); fibronectin (E1). **F1)** Low expression of Oct3/4 in EP; **A2–F2)** Flow cytometry showing LP of IDPSCs, which expressed same markers as EP. **F2)** Higher expression of Oct3/4 in LP, than in F1. **A3–F3)** Immunofluorescence of LP of IDPSCs using same markers as in (A2–E2). **F3)** Nuclear localization of Oct3/4 can be observed. A3–F3) Epi-fluorescence, nuclei stained with DAPI (blue). Scale bars: A3, B3, E3, F3 = 5 µm; C3, D3 = 10 µm.

EP and LP were characterized using several markers, which were employed in our previous original study [4], such as SH2/CD105, SH3/CD73 (MSCs markers) and Oct3/4 (ES cells marker). Additionally, expression of MSCs markers such as vimentin, nestin, fibronectin has been evaluated in both EP and LP. The reason of choosing these markers will be explained in discussion section. Representative Figure 3 (A1–E1, A2–E2) showed that all MSC markers were expressed in both populations (EP and LP) and slightly declined in LP after six month of multiple DP transfer (Figure 3A2–E2). A percentage of IDPSCs, which showed positive immunostaining for these markers, was evaluated by flow cytometry and was 99.10% to EP and 96.60% to LP for SH2/CD105; 99.60% to EP and 98.40% to LP for SH3/CD73; 97.76% to EP and 94.56% to LP for nestin; 99.45% to EP and 95.60% to LP for vimentin; 97.10% to EP and 96.30% to LP for fibronectin (Figure 3A1–E1, A2–E2). Interestingly, that in this particular IDPSC population (which is not a rule) a very low percentage of Oct3/4 positive cells ∼0.75% was observed in EP, which increased to ∼10.03% in LP (Figure 3F1, F2).

Flow cytometry data have been confirmed by cells immunostaining using antibodies against the same MSC and ES cell markers. Their expression was observed in both EP and LP of IDPSCs. In Figure 3, the expression of MSC markers in LP is presented (Figure 3A3–E3). As expected, Oct-3/4 protein expression was observed in the cell nuclei (Figure 3F3). The expression of all these markers in LP was similar to EP (data not shown).

### Culture Media Influence EP and LP Growth Rate and Gene Expression

**Figure 4:**
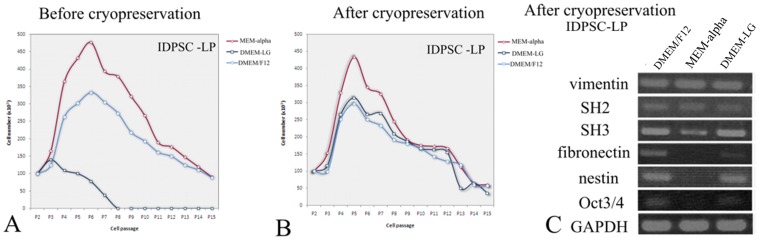
Proliferation rate and gene expression of IDPSCs after cultivation in three distinct culture media. A ) Proliferation curve of LP before cryopreservation; **B**) Proliferation curve of LP after cryopreservation. **C**) Gene expression of LP after cryopreservation.

Proliferative capacity of EP and LP of IDPSCs before and after cryopreservation was studied using three different culture media: DMEM/F12, DMEM-LG, and MEM-alpha. Starting from P2, non-cryopreserved cells were harvested following enzymatic dissociation and counted daily during 15 consecutive passages. IDPSCs cultured in DMEM/F12 and MEM-alpha medium, presented constant proliferative rate during initial passages, which achieved their peak growth = 5±2 (Figure 4A, Table 1). Based on growth curves presented in Figure 4, statistical analyses were performed considering the cell number from passage 3 to 7 (Table 1). Using the same parameters, proliferative rate of EP and LP cultivated in DMEM/F12 and MEM-alpha media were evaluated after thawing and showed similar proliferative potential, when compared with those before cryopreservation (Figure 4B, Table 1). Non-cryopreserved EP and LP of IDPSCs cultured in DMEM-LG presented spontaneous differentiation into osteogenic lineage (data not shown) and demonstrated rapid decrease of proliferative potential (Figure 4A, Table 1). Interestingly, EP and LP of IDPSCs, cultured in DMEM-LG after thawing, maintained their proliferative state (Figure 4B, Table 2). DMEM/F12 and MEM-alpha media did not induce any spontaneous differentiation in non-cryopreserved and cryopreserved EP and LP of IDPSCs.

**Table 1:** Number of IDPSCs cultured in three different growth media and at different passages before and after cryopreservation.

Cell line	Growthmedia	Passage number													
		2	3	4	5	6	7	8	9	10	11	12	13	14	15
Beforecryopreservation	MEM-alpha	100	165.5	364	431.5	476.5	394	378.5	321	266.5	189	176.5	147.5	119.5	90.5
	DMEM-LG	100	139.5	109.5	100	78	38.5	a	a	a	a	a	a	a	a
	DMEM/F12	100	125	262.5	302	333	305.5	273.5	218	193	161.5	149.5	124.5	110.5	89
Aftercryopreservation	MEM-alpha	100	153	329	433.5	345	325.25	245	191.5	175.5	172.5	166.5	112.5	64.6	60.5
	DMEM-LG	100	115.5	265	314	266	268.50	209	186.5	165.5	162	154.5	50	64.5	35.75
	DMEM/F12	100	99	251.5	296.5	250.5	232.5	191	180.5	166.5	142	128	117.5	59.5	57

a DMEM-LG did not support cell growth.

**Table 2:** Cell number (x10^3^) of IDPSCs (passage range P3–P7) cultured in different growth media before and after cryopreservation.

Cryopreservation	Growth media		
	MEM-alpha	DMEM-LG	DMEM/F12
Before	317.15±101.81^a^	245.80±75.70^a^	226.00±74.82^a^
After	366.30±119.88^a^	93.10±37.68^b^	265.60±82.52^a^

a Values are mean±standard deviation, n = 5. Means followed by the same letter are not statistically different (Tukey, p>0.05).

The gene expression pattern of pluripotent ES cell and MSC markers were analyzed by RT-PCR in EP and LP after thawing. Both were cultured in different basal media (DMEM/F12, MEM-alpha, and DMEM-LG) for seven passages. Overall, both EP and LP showed similar expression pattern of vimentin, SH2/CD105 and SH3/CD73 (Figure 4C). Similar expression pattern was observed to fibronectin, nestin and Oct3/4, when IDPSCs were cultured in DMEM/F12 and DMEM-LG. However, it was distinct when cultivated in MEM-alpha, in which three of these genes (fibronectin, nestin and Oct3/4) did not show any expression (Figure 4C).

### Chondrogenic and Myogenic Differentiation

Multipotential capacity of IDPSCs was reported elsewhere [4]. Therefore, only two differentiation assays were chosen to demonstrate their differentiation capacity.

**Figure 5:**
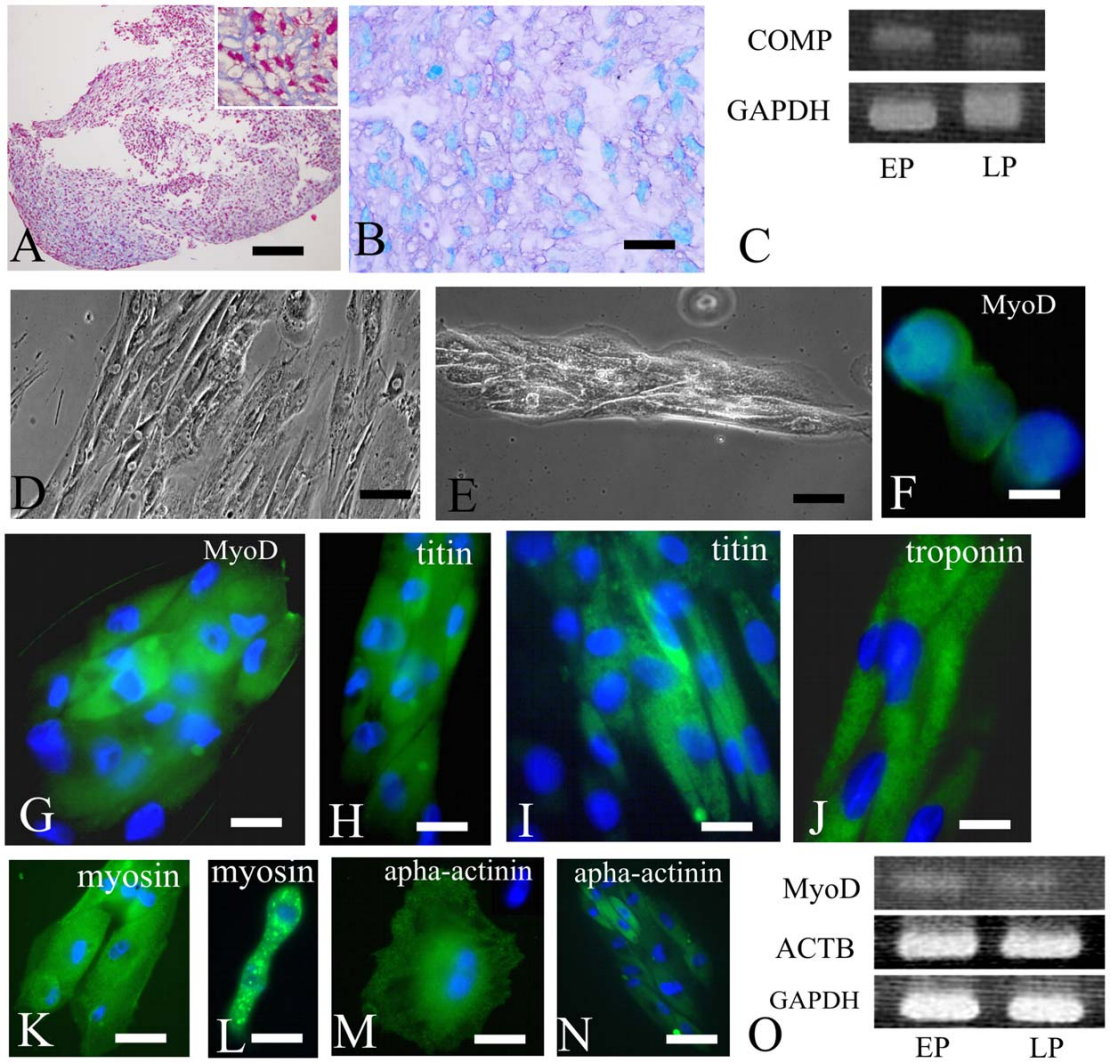
*In vitro* differentiation potential of IDPSCs. **A–C**) Chondrogenic differentiation. **A**) Pellet culture: collagen fibers intensively stained by Massoǹs thrichrome. Inset: same as in (A) high magnification. **B**) The proteoglycans presence was revealed by Toloudine blue staining. **C**) RT-PCR shows the expression of COMP gene in EP and LP of IDPSCs. Housekeeping gene GAPDH is used as control. **D–O**) Myogenic differentiation. **D, E**) Morphological aspect showing stages of muscle fibers formation. **F**) Nuclear expression of MyoD1 protein in LP of IDPSCs-derived myocyte-like cells. **G**) Myosac composed by MyoD1 positive cells. **H, I**) Titin protein expression in LP of IDPSCs-derived myotubes. **J**) Expression of troponin I in Z-bands of myofibers. **K**) Myosin protein expression. **L**) Very small, satellite-like cells, showing positive myosin immunostaining. **M**) Binuclear cell positive for alpha-actinin (spot-like labeling). **N)** Fused myotubes, which deferentially express alpha-actinin protein. **O**) RT-PCR shows the expression of MyoD1 and ACTB genes in EP and LP of IDPSCs. Housekeeping gene GAPDH is used as control. A, B, D, E) Light Microscopy; F-N) Epi-fluorescence, nuclei stained with DAPI (blue). Scale bars: A = 200 µm; B = 20 µm; D = 50 µm; E, N = 10 µm; F–M = 5 µm.

At day 21 after induction of chondrogenic differentiation, IDPSCs demonstrated the formation of an extracellular cartilage matrix which was intensively stained by Massoǹs trichrome (Figure 5A, Inset). Toluidine blue staining was used to detect essential cartilage matrix proteins such as proteoglycans (Figure 5B). IDPSCs maintained in basal culture medium (control) did not form any cell pellet (data not shown). Additionally, chondrogenic differentiation was confirmed by the expression of COMP (Cartilage Oligomeric Matrix Protein) gene, which encodes a pentameric non-collagenous matrix protein that is mainly expressed in articular cartilage. The expression of COMP was observed in both EP and LP of IDPSCs (Figure 5C). It is important to highlight that chondrogenic differentiation of IDPSCs was uniform even in the absence of TGF-β, which is known to be a strong inductor of chondrogenesis in bone marrow-derived MSCs [19], [20].

Following myogenic differentiation, IDPSCs showed typical cells elongation and fusion leading to small myotubes formation at day 7 (Figure 5D). At day 21, this cell fusion was obvious and most of the cells formed small myofibers (Figure 5E). MyoD transcription factor, which is a master regulatory gene of skeletal muscle differentiation, as expected, was expressed in IDPSC-derived myoblasts in nucleus or in perinuclear space following immunostaing using anti-MyoD1 antibody (Figure 5F). These myoblasts further form myosacs and MyoD1 protein was observed in the cytoplasm of these more mature cells (Figure 5G). Titin is the third most abundant skeletal muscle filamentous protein that forms a separate myofilament system in both skeletal and cardiac muscle. It was expressed in IDPSC-derived muscle cells at more advanced stages of differentiation (Figure 5H). Some titin negative cells were also observed (Figure 5I).

Troponin I is a protein responsible for immobilizing the actin-tropomyosin complex in place. The expression of this protein was visualized in more mature myofibers-derived from IDPSCs (Figure 5J). Human specific anti-actinin and anti-myosin antibodies reacted positively with differentiated IDPSCs (Figure 5K–N). Myosin positive immunostaining was observed in myofibers (Figure 5K) and also in differentiated small cells, which presented spot-like immunolabeling (Figure 5L). Singular binuclear differentiated IDPSCs were alpha-actinin positive (Figure 5M). This marker showed differential expression pattern within myosacs: some cells were strongly positive, while others presented only shadow-like immunostaining (Figure 5N). RT-PCR was used to verify the expression of MyoD1 and ACTB (Beta cytoskeletal actin) genes during IDPSCs myogenic differentiation. Both genes were found to be expressed in EP and LP (Figure 5O). EP and LP of IDPSCs showed similar chondrogenic and myogenic differentiation before and after cryopreservation. Control culture of IDPSCs did not present any signals of myogenic differentiation (data not shown).

### Expression of MSCs and ES Cells Markers in DP

**Figure 6:**
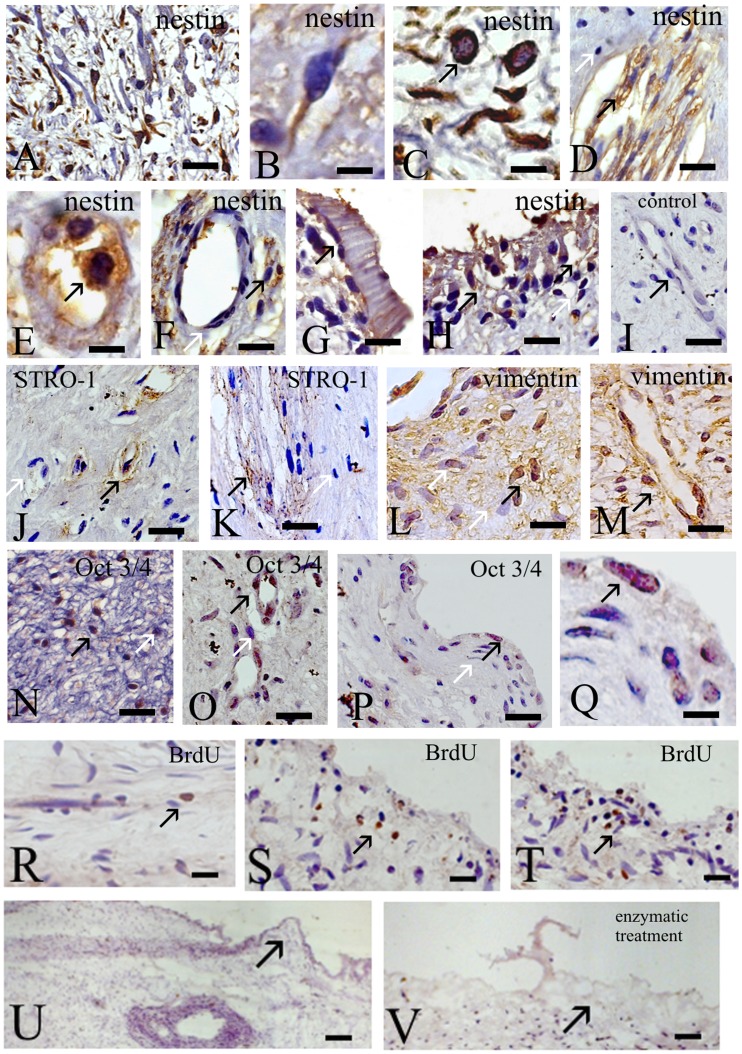
Expression of nestin, STRO-1, vimentin, Oct3/4 and BrdU in DP. **A–H**) Nestin expression. **A–C**) Cell rich zone. **A**) Multiple nestin positive cells can be observed. Here and below black arrows indicate immunopositive, while white arrows - immunonegative cells. **B**) Supposedly undifferentiated MSC shows nestin cytoplasm localization. **C**) Nestin positive cells with two distinct morphologies round epithelial-like (ES-like) and fibroblast-like cells. **D–F**) Cell free zone. **D**) Nestin showing intermediate filament staining in nerve plexus. **E**) Small capillary with two intensively stained nestin positive cells. **F**) Same as in (E) with nestin positive cells in lateral of capillary (arrow). **G, H**) Odontoblastic layer. Nestin positive obontoblasts (G, H) can be observed. **I**) Negative control: only secondary antibody was used. **J, K**) Cell free zone. **J**) STRO-1 positive cells within capillaries (perivascular niche). **K**) Very poor STRO-1 immunostaining was observed within nerve plexus. **L, M**) Vimentin positive (black arrows) cells localization in cell rich (L) and cell free (M) zones. **N–Q**) Oct3/4 positive cells localization in cell rich (N) and cell free (O–Q) zones. **R–T**) BrdU immunostaining of DP. **R)** DP just after plating in culture medium. **S)** 48 hours after *in vitro* cultivation. **T)** 72 hours after *in vitro* cultivation. **U–V)** DP without (U) and with (V) enzymatic treatment. **V)** External cell layer of DP is destroyed by such treatment. A–V) Light Microscopy. Scale bars: A, D, F–P, R–T = 20 µm; B, C, Q = 5 µm; U, V = 50 µm.

We observed that both EP and LP of IDPSCs cultured *in vitro* are rich in nestin and vimentin positive cells (Figure 3C1–C3). Therefore, we attempted to identify the exact niche of nestin, vimentin and Oct3/4 positive cells within DP using immunohistochemical assay (Figure 6A–Q). Nestin positive cells were found in all zones of DP: in cell rich zone (innermost pulp layer which contains fibroblasts and undifferentiated mesenchymal cells) (Figure 6A–C), in cell free zone, nestin expression was observed in both capillaries and nerve networks (Figure 6D–F); as well as in odontoblastic layer (outermost layer which contains odontoblasts and lies next to the predentin and mature dentin) (Figure 6G, H). Nestin positive cells in cell rich zone showed fibroblast-like as well as ES-like cell morphologies (Figure 6B, C). In cell free zone, nestin protein was found to be expressed in intermediate filaments in the cells from plexus of nerves (Figure 6D), as well as nestin positive cells were embedded in the wall of small capillaries (Figure 6E) and in adjacent regions of these capillaries (Figure 6F). In odontoblastic layer, several round ES cell-like and large columnar cells were also nestin positive (Figure 6G, H). STRO-1 antibody was used as control, once this marker was described to be specific for stem cells/pericytes from DP. The expression of this marker was mainly observed in small capillaries and middle size blood vessels (Figure 6J), as well as in plexus of nerves in the cell free zone (Figure 6K). We also verified localization of vimentin expressing cells in DP. As expected, vimentin positive cells were localized in capillaries and in innermost pulp layer, locals where nestin positive cells were also found (Figure 6L, M). Once low percentage of Oct3/4 positive cells, which increased with time of DP *in vitro* cultivation, was observed in IDPSCs, the expression of this protein in DP was also checked. Strong expression of Oct3/4 in the cells nuclei, localized in DP capillaries and in innermost pulp layer can be observed (Figure 6N–Q).

### Localization of BrdU Positive Cells in DP during *in vitro* Cultivation

In order to understand continuous process of IDPSCs generation, DP was treated with BrdU just after extraction and *in vitro* plating (Figure 6R–T). After 6 h, only few anti-BrdU antibody positive cells were found in the central part of DP (Figure 6R). After 48 h, BrdU positive cells were observed in the periphery of DP (Figure 6S), while after 72 h, it seems, that BrdU positive cells increased in number and were also found in the periphery of DP in the apical part, close to IDPSCs outgrowing zone (Figure 6T). Morphological aspect of DP with and without enzymatic treatment (collagenase/dispase) was compared (Figure 6U, V). DP, without any treatment, maintains their integrity especially in the region where BrdU positive cells were observed (Figure 6U), while after enzymatic digestion this region was destroyed (Figure 6V).

## Discussion

Stem cells reside in a quiescent state within all organs of organism in their special niche and they start to proliferate and to migrate when their niche experiences changes [21]–[24]. Thus, culture of adult SC niche may provide harvesting of SCs at high scale. We developed a method of long-term DP culture, which allowed harvesting of large quantities of IDPSCs (Figure 2). Mechanical transfer, before and after DP cryopreservation, can also be performed as long as it is necessary and in our experience, it may stop due to, e.g. occasional DP explant contamination. IDPSCs are uniform in respect of morphology (light microscopy and TEM analyzes) (Figure 1A–F) and karyotype of cells remained unchanged (Figure 1G). These cells express high percentage of SC markers such as SH2/CD105, SH3/CD73, nestin, vimentin, fibronectin and low percentage of Oct3/4 (Figure 3A1–F3) without losing their original properties [25].

To date, standardized protocol of SCs culture from DP is not available. Therefore, we directed our study to optimization culture medium conditions for scaling-up of IDPSCs. DMEM-LG, MEM-alpha and DMEM/F12 are culture media which are commonly used for the isolation and expansion of MSCs [26]. In the present work, we verified the effect of DMEM-LG, MEM-alpha and DMEM/F12 on proliferation rate and gene expression pattern of IDPSCs (Figure 4). These analyses indicated that MEM-alpha and DMEM/F12 were the most appropriate media for the isolation and long-term expansion of IDPSCs (Figure 4A, B, Table 2); distinct gene expression patterns were observed between these media (Figure 4C). DMEM-LG was not efficient for the isolation, but it was able to support long-term expansion of these cells after their cryopreservation (Figure 4A, B, Table 1). It seems that our present data (Figure 4, Table 1) are in contrast with previous observations, which showed IDPSCs exponential growth following multiple passages [4]. However, in the present study, cells were counted daily, while in previous study, passages were performed every 3–4 days. Therefore, enzymatic treatment used daily seems to have hampered the IDPSCs.

Analysis of differentiation potential toward chondrogenic and myogenic lineages evidenced high differentiation potential of LP and EP of IDPSCs (Figure 5A–O) comparable to those described previously [4]. IDPSCs showed similar chondrogenic and myogenic differentiation before and after cryopreservation (data not shown) as was reported previously for other SCs from DP [27], [28]. Cryopreservation process preserves the proliferative and differentiation capacity of IDPSCs and, thus, allows the opportunity to bank these valuable DTSCs [29].

Recently, new populations of DTSCs were isolated and were shown to be distinct from DPSC (Dental Pulp Stem Cells from permanent teeth)/SHED (Stem Cells from Human Exfoliated Deciduous teeth) [1], [2], [4], [30]–[35]. As reported in several original publications, DPSC/SHED are supposed to be pericytes, which are isolated from perivascular niche [36], [37]. To delineate the anatomic localization of IDPSCs inside the pulp, we performed *in situ* analysis using markers of MSCs and ES cells. Our study suggested that DP has multiple SC niches, which are localized in capillaries and nerve networks in cell free zone (Figure 6D–F); in innermost pulp layer in cell rich zone (Figure 6A–C) and in outermost layer, which contains odontoblasts (Figure 6G, H). All these niches are rich of nestin positive cells [38], which can present fibroblast-, epithelial- and odontoblast-like morphologies (Figure 6A–I). In accordance with our finding, recently, rare quiescent multipotent nestin positive MSCs were found in bone marrow in association with hematopoietic SCs and adrenergic nerve fibers. However, bone marrow derived colonies of MSCs cultured *in vitro* exhibit a low percentage (∼4%) of such nestin positive cells [39]. Meanwhile, STRO-1 positive cells localization in DP, as expected, is restricted to endothelium (Figure 6J), albeit very weak positive immunostaining was observed in nervous plexus (Figure 6K). Vimentin showed distribution within DP similar to nestin, however, a smaller amount of cells positive for this markers can be observed (Figure 6L, M). Overall, these data suggest that IDPSCs constitute a mixed population of both MSCs and epithelial SCs, among which the pericytes (SHED) are also present. Surprisingly, multiple Oct3/4 positive cells were found in DP (Figure 6N–Q), showing the appropriate nuclear localization (Figure 6Q). After isolation, IDPSCs did not show as high as expected percentage of Oct3/4 positive cells. Previously, we succeeded to isolate IDPSCs population, which contained ∼20% of these cells. We supposed that in the pulp, Oct3/4 positive cells are highly pluripotent and of epithelial type. When these cells start to migrate, they undergo epithelial-mesenchymal transition, which leads to a decrease or lost Oct3/4 expression. Thus, another method can be developed in order to isolate naïve Oct3/4 positive cells from their niche in DP.

DP is capable to produce long-term culture of SCs; however the process of such ability is unknown. BrdU incorporation into DP demonstrates that, as expected, only very rare BrdU positive cells were observed just after DP extraction and plating. Following further cultivation, such cells increased in number and are located in the periphery of DP. It is plausible to suggest, that the isolation of DP stimulates the mechanisms leading to SC proliferation and migration (Figure 6R–T). In contrast to previous original report [1], which used the method of DP enzymatic dissociation, we choose DP explant as a main method of SCs isolation [4]. Comparative morphological analysis demonstrate that prior SCs isolation enzymatic treatment is not recommended for DP which may destroy future “niche” of SCs (Figure 6U, V).

In conclusion, our method provides the isolation of a high purity SC population in substantial quantities. It is based on natural (intrinsic) mechanisms of SCs activation similar which occur during tissue trauma or injury, when in response to their damage, quiescent SCs are activated. Our method can be applied to isolate SCs from single and multiple niches from any type of adult tissues, such as bone marrow, adipose tissue, umbilical cord, muscles, skin and others. This protocol diminishes a probability of occurrence of spontaneous genomic mutations and eventual karyotype abnormalities, which may arise during multiple passages in SCs. This method is simple, does not requires long time DP preparation, guarantees sterility (DP can be transferred using sterile instruments or even pipette) and avoids any type of SCs selection, which is undesirable for future clinical applications.

## Materials and Methods

### Human Dental Pulp Extraction and Cell Culture

The investigation was approved by the Ethical Committee of the Federal University of Sao Paulo (Protocol N° 0139/10). Human DP was extracted from ten deciduous teeth of ten healthy subjects (range 6–9 years) following previously established protocol [4]. All patients agreed to participate of this study as well as, their next of kin, carers or guardians on the behalf of the minors/children participants signed a written informed consent. Next, DP was gently rinsed in phosphate-buffered solution (PBS) (Invitrogen, Carlsbad, CA, USA), slightly dissected and placed into 35 mm plastic tissue culture dishes (Corning Inc., Corning, NY, USA). Tissue explants were cultured in Dulbecco’s-modified Eagle’s medium (DMEM)/Ham’s F12 (DMEM/F12, Invitrogen Corporation - Carlsbad, CA, USA) supplemented with 15% fetal bovine serum (FBS, Hyclone, Logan, Utah, USA), 100 units/ml penicillin, 100 µg/ml streptomycin, 2 mM L-glutamine, and 2 mM nonessential amino acids (all from Invitrogen) in a 5% CO_2_ humid atmosphere at 37°C. After a period of 3 or 4 days, fibroblast-like cells were generated from adherent explants. Explants were transferred to another Petri dish under the same culture conditions; this procedure was repeated several times (Figure 2). Fibroblast-like cells growing in monolayer were further washed twice with PBS and subjected to 0.5 g/L trypsin and 0.53 mmol/L Ethylenediamine tetra-acetic acid (EDTA) (Invitrogen) for 3 to 5 minutes at 37°C. Passage 1 was counted after the first enzymatic digestion. Trypsin action was inactivated by culture medium supplemented with 10% FBS and cells (∼5×10^5^) were placed into 25 cm^2^ cell culture flask (Corning). This subculturing was performed each 3–4 days and the culture medium was changed daily. For cryopreservation, 90% FBS and 10% dimethylsulfoxide (DMSO) (Sigma, St. Louis, Mo., USA) were used as freezing medium. Frozen cells were maintained in sealed vials at −196°C.

### Karyotype Analyses

Karyotyping of subconfluent EP and LP of IDPSCs cultured in DMEM/F12 medium (Invitrogen) was performed at passage 3. Before harvesting, demecolcine (Sigma) at a final concentration of 0.1 µg/ml was added for 1 hour. Cells were harvested, washed in PBS and resuspended in 0.5 ml of medium and mixed with 0.075 M KCl (Sigma) to a volume of 10 ml. After incubation for 20 minutes at room temperature, cells were centrifuged at 400 g for five minutes and the pellet fixed in 5 ml three times (3∶1) of cold methanol/acetic acid (Sigma). Three drops of cell suspension were fixed per slide. For chromosome counting, slides were stained in Giemsa for 15 minutes and; *>*200 cells were analyzed per cell line and reported on a Zeiss II microscope (Zeiss, Jena, Germany) according to the International System for Human Cytogenetic Nomenclature.

### Transmission Electron Microscopy (TEM)

For TEM, EP and LP of IDPSCs were fixed in 2.5% glutaraldehyde (Sigma) for 48 h, post-fixed in 1% phosphate-buffered osmium tetroxide solution (pH 7.4) (Sigma) for 2 h at 4°C and embedded in Spurr’s Resin (Sigma). Ultrathin sections were obtained using an automatic ultramicrotome (Ultracut R, Leica Microsystems, Germany). Sections were double-stained with uranyl acetate (Sigma) and lead citrate (Sigma) (2% and 0.5%, respectively) and analyzed using TEM (Morgagni 268D, FEI Company, The Netherlands; Mega).

### Antibodies and Immunophenotyping

EP and LP of IDPSCs immunophenotyping was based on immunofluorescence and flow-cytometry analyses performed by using anti-human specific antibodies (vimentin, nestin, fibronectin, Oct3/4 (all from Santa Cruz Biotechnology, Santa Cruz, CA, USA), CD105/SH-2 and CD73/SH-3 (both from Case Western Reserve University, OH, USA). FITC-conjugated secondary antibodies (Chemicon, Temecula, CA, USA) were used and respective isotype matched controls. Immunofluorescence were analyzed using these aforementioned antibodies after cell fixation in 4% paraformaldehyde (Sigma) in PBS and permeabilization in 0.1% Triton X-100 (Sigma) in PBS. IDPSCs were incubated with 5% bovine serum albumin (BSA, Sigma) diluted in PBS for 30 minutes and further incubated for 1 h at room temperature with FITC-conjugated goat anti-mouse or anti-rabbit immunoglobulin (Chemicon) at a final dilution of 1∶500 in PBS (Invitrogen). Microscope slides were mounted in Vectashield mounting medium with 4′,6-Diamidino-2-phenylindol (DAPI, Vector Laboratories, Burlingame, CA) and immunofluorescence was detected using a Carl Zeiss Axioplan fluoromicroscope (LSM 410, Zeiss, Jena, Germany) or Nikon Eclipse E1000 (Nikon, Kanagawa, Japan). Digital images were acquired with CCD camera (Applied Imaging model ER 339) and the documentation system used was Cytovision v. 2.8 (Applied Imaging Corp. - Santa Clara, CA, USA). Flow-cytometry was performed using EP and LP of IDPSC at passage 3. Cells were detached by using a 10 min treatment at 37°C with PBS 0.02% EDTA, pelleted (10 min at 400 g) and washed in 0.1% BSA in 0.1 M PBS at 4°C. Next, cells at a concentration of 10^5^ cells/ml were stained with saturating concentration of aforementioned antibodies (10 µl). After 45 minute incubation in the dark at room temperature, cells were washed three times with PBS and resuspended in 0.25 ml of cold PBS. Flow-cytometry analysis was performed on a fluorescence-activated cell sorter (FACS; Becton, Dickinson, San Jose, CA) using the CELL Quest program (Becton, Dickinson). The flow cytometry and/or immunofluorescence analyses were repeated with all samples (n = 10), and one representative experiment is presented. All experiments have been done in triplicate and furthermore were repeated several times.

### Cell Growth Rate

To evaluate the effect of different culture media on cell growth, freshly isolated and the same IDPSC frozen–thawed were equally divided in three groups (DMEM/F12, DMEM low-glucose (1000 mg/ml; DMEM-LG) and Minimum Essential Medium (MEM) Alpha Medium (MEM-alpha). All media (Invitrogen) were supplemented with 15% FBS (Hyclone), 100 units/ml penicillin, 100 µg/ml streptomycin, 2 mM L-glutamine, and 2 mM nonessential amino acids (all from Invitrogen). Cells were seeded at a density of 10^5^/ cm^2^ counted for at least fifteen consecutive days to evaluate the growth rate and the effect of cryopreservation. We also verified the capacity of DP tissue explant to produce IDPSC after consecutive rounds of cryopreservation and thawing. All experiments were performed in triplicate.

### Data and Statistical Analysis

Growth curves were constructed using data from cell lines, passage number (P2 to P15), cryopreservation and growth medium. Cell number data were analyzed by using two-way analysis of variance (“cryopreservation” and “growth medium”) complemented by Tukey post hoc multiple comparison tests. The significance level was set at 5% (SPSS 19.0, Chicago, IL, USA).

### RNA Extraction and Reverse Transcription-polymerase Chain Reaction (RT-PCR)

EP and LP of IDPSCs were cultivated during seven passages in three distinct media (DMEM/F12, MEM-alpha, and DMEM-LG). To evaluate the effect of these different culture media on gene expression, total RNA was extracted using Trizol (Invitrogen): IDPSCs were washed in PBS and RNA extraction was performed according to manufactures instructions. cDNAs were synthesized from 1 µg of total RNA reverse transcribed with the RevertAid M-MuLV Reverse Transcriptase and oligo (dT) (Fermentas Life Science, Amherst, NY, EUA) according to the manufactures instructions. The final concentrations of reagents were: 20 µl of PCR reactions were prepared with 2 µl cDNA, 0,2 µM of each primer, 1 unit of Taq DNA Polymerase, 0,2 µM of dNTPs, 1,5 mM of magnesium chloride and buffer Taq DNA Polymerase (Fermentas Life Science). Primer sequences (forward and reverse), and the lengths of amplified products are summarized: Nestin FW 5′- CTCTGACCTGTCAGAAGAAT-3′, and RV 5′-GACGCTGACACTTACAGAAT-3′ (302 bp/54°C); Vimentin FW 5′-AAGCAGGAGTCCACTGAGTACC-3′, and RV 5′-GAAGGTGACGAGCCATTTCC-3′ (205 bp/55°C); Fibronectin FW 5′-GGATCACTTACGGAGAAACAG-3′, and RV 5′-GATTGCATGCATTGTGTCCT-3′ (386 bp/56°C); OCT3/4 FW 5′-ACCACAGTCCATGCCATCAC-3′, and RV 5′-TCCACCACCCTGTTGCTGTA-3′ (120 bp/61°C); SH2/CD105 FW 5′- TCTGGACCACTGGAGAATAC-3′, and RV 5′-GAGGCATGAAGTGAGACAAT-3′ (171 bp/56°C); SH3/CD73 FW 5′-ACACGGCATTAGCTGTTATT-3′, and RV 5′-AGTATTTGTTCTTTGGGCA-3′ (391 bp/56°C). For chondrogenic and myogenic differentiation, following primer sequences were used: COMP FW 5′-CCGACAGCAACGTGGTCTT-3′, and RV 5′-CAGGTTGGCCCAGATGATG-3′ (91 bp/53°C); ACTB FW 5′-TGGCACCACACCTTCTACAATGAGC-3′, and RV 5′ GCACAGCTTCTCCTTAATGTCACGC-3′ (395 bp/59°C); MYOD1 FW 5′-GCCGCCTGAGCAAAGTAAATGAGG-3′, and RV 5′-TAGTCCATCATGCCGTCGGAGC-3′ (280 bp/53°C). GADPH gene FW 5′- ACCACAGTCCATGCCATCAC-3′, and RV 5′-TCCACCACCCTGTTGCTGTA-3′ (463 bp/61°C) was used as control. Undifferentiated IDPSC were examined as negative control for differentiation specific primers. PCR reactions were performed under the following conditions: 1 cycle at 94°C for 5 minutes, followed by 35 cycles at 94°C for 1 minute, annealing temperature for 1 minute, and 72°C for 1 minute. Amplified products were resolved by electrophoresis on a 1.5% agarose gel (Sigma) and visualized using ethidium bromide (Sigma) staining.

### Differentiation Assays

#### Chondrogenic differentiation

The differentiation was performed using pellet culture technique [19], [20]. EP and LP of IDPSCs populations from sub confluent cultures (passage 3) were released by 0.5 g/L trypsin and 0.53 mmol/L EDTA, counted and used to generate micromass culture. Briefly, 4×10^6^ cells were centrifuged at 500 g in 15 ml polypropylene conical tubes (Corning) and the resulting pellets were cultured for 4 weeks. Control cultures were grown in a serum-free chemically defined medium consisting of DMEM, high-glucose (4,500 mg/L; DMEM-HG) (Invitrogen) supplemented with 6.25 µg/ml insulin, 6.2 µg/ml transferrin, 6.25 µg/ml selenious acid, 5.33 µg/ml linoleic acid (ITS, Premix, BD, USA) and 1 mM sodium pyruvate (Invitrogen). To induce chondrogenic differentiation, control medium was supplemented with 0.1 µM dexamethasone and 50 µg/ml ascorbate-2-phosphate (both from Invitrogen), without transforming growth factor beta (TGF-β). Cultures were incubated for 4 weeks at 37°C in a humid atmosphere containing 5% CO_2_; the medium was changed every day. Cell aggregates were harvested at 4 weeks for RT-PCR, with primer sequences aforementioned, and were also fixed for histology.

#### Muscle differentiation

EP and LP of IDPSCs at the passage 3 were seeded at a concentration of 5000 cells/cm^2^. Control cultures were grown in a serum-free chemically defined medium consisting of DMEM-HG (Invitrogen) supplemented with 10% FBS (Hyclone). To induce myogenic differentiation, control medium was supplemented with 50 µM hydrocortisone (Sigma) and 5% horse serum (Invitrogen). Cultures were incubated for 21 days at 37°C in a humid atmosphere containing 5% CO_2_; the medium was changed every three days. Then, cultured cells were fixed for histology and for immunohistochemistry with antibodies that recognize human muscle proteins. Aforementioned primers sequences were used to detect myogenic differentiation of IDPSCs.

### Histological Analyses

#### Pellet culture analysis

Cell aggregates were fixed in 4% formaldehyde in PBS for 40 minutes and embedded in paraffin (Sigma). Paraffin sections were stained with Masson’s trichrome (Sigma) and Toluidine Blue (Sigma) and analyzed using transmitted and polarized light microscopy Nikon Eclipse E1000 (Nikon), digital images were acquired by CCD camera (Applied Imaging).

#### Muscle analysis

Slices were fixed in 4% paraformaldehyde diluted in PBS for 40 min and analyzed using transmitted and polarized light microscopy Nikon Eclipse E1000 (Nikon), digital images were acquired by CCD camera (Applied Imaging).

### Immunohistochemistry

Troponin I (Chemicon), titin (Chemicon), alpha-smooth muscle actinin (Sigma), MyoD1 (Chemicon) and sarcomeric myosin (Chemicon) proteins were analyzed using specific antibodies. First, cells were fixed in 4% paraformaldehyde diluted in PBS and permeabilized in 1% Triton X-100 diluted in PBS. EP and LP of IDPSC that were submitted to myogenic differentiation were incubated in 5% BSA diluted in PBS for 30 min and incubated for 1 h at room temperature with FITC-conjugated goat anti-mouse or anti-rabbit immunoglobulin (Chemicon) at a final dilution of 1∶500 in PBS. Microscope slides were mounted in Vectashield mounting medium with DAPI and immunofluorescence was detected using a Carl Zeiss Axioplan fluoromicroscope (Zeiss) or Nikon Eclipse E1000 (Nikon), digital images were acquired by CCD camera (Applied Imaging) and the documentation system used was Cytovision v. 2.8 (Applied Imaging Corp).

### Pulp Tissue Experiments

#### Tissue specimens

Ten deciduous teeth of ten healthy subjects (range 5–10 years), free of caries and restorations, were extracted and initially rinsed in PBS. Seven pulps were gently removed and fixed in 10% formalin solution for 48 h. The specimens were embedded in paraffin blocks and sections of 10 µm were obtained. The other three pulps were gently rinsed in PBS and sliced. Each slice was placed in different culture dish. Next, 5-bromo-2′-deoxyuridine (BrdU, Sigma) was added directly into basal culture medium. First pulp slice was fixed and processed after 6 h of treatment with BrdU. In the second culture dish with pulp slice, BrdU was added after 42 h and in the third - after 66 h. After 6 h of treatment with BrdU, all slices were fixed in 10% formalin solution for 48 h. The specimens were embedded in paraffin blocks and sections of 10 µm were obtained. All above specimens were treated by immunohistochemical methods.

### Immunohistochemical

Paraffin sections of 10 µm were deparaffinized and then hydrated. Endogenous peroxidase activity was measured by incubating the sections for 30 min in a 0.1% solution of hydrogen peroxide (Sigma). For antigen retrieval, sections were incubated with trypsin for 10 min at 37°C. To inhibit nonspecific antigen binding, sections were incubated with blocking serum (5% fetal calf serum, Invitrogen) for 10 min. Sections were then incubated for 12–16 h with the primary antibody in a moist chamber at 4°C. Primary antibodies were the same used in immunophenotyping of IDPSCs and additionally anti-human STRO-1 (Santa Cruz) and mouse anti-BrdU IGg (Chemicon). The optimal dilution of the primary antibody was found to be 1∶10. Slides were again rinsed with PBS and then incubated with biotinylated secondary antibody (DAKO, Glostrup, Denmark) in 1∶200 dilution for 30 min. The samples were washed with PBST (PBS with 0.1% of Tween 20) and incubated with StrepABComplex/HRP (DAKO) at 1∶100 dilution for 30 min. After one more wash with PBST, the colour was revealed by the chromogen 3 (3-diaminobenzidine DAB Kit, Zymed Laboratories, Inc.) for 5 min, followed by PBST washing, nuclear counterstaining with Harris haematoxylin for 45 s, dehydrated and mounted in Permount. Observation of the sections was conducted using a Carl Zeiss Axioplan fluoromicroscope (Zeiss). Negative control sections were treated identically, except the primary antibody which was substituted by PBS.
